# 2275. No "One-Size-Fits All": Chronic "Carryover" Diagnoses Dilute Antibiotic Prescribing Metrics in Primary Care

**DOI:** 10.1093/ofid/ofad500.1897

**Published:** 2023-11-27

**Authors:** Mary Smith, Marten Hawkens, Chananid Laikijrung, Emily Mui, Thomas Leung, Alex Zimmet, David R Ha, Marisa Holubar

**Affiliations:** Stanford Healthcare, Stanford University School of Medicine, Santa Clara, California; Stanford Healthcare, Stanford University School of Medicine, Santa Clara, California; Stanford Healthcare, Stanford University School of Medicine, Santa Clara, California; Stanford Health Care, STANFORD, CA; Stanford Healthcare, Stanford University School of Medicine, Santa Clara, California; Stanford Healthcare, Stanford University School of Medicine, Santa Clara, California; Stanford Health Care, STANFORD, CA; Stanford University School of Medicine, Stanford, CA

## Abstract

**Background:**

International Classification of Diseases, Tenth Revision (ICD-10) encounter coding is used to identify sub-optimal outpatient antibiotic use, but the utility of a popular billing data based metric, antibiotic prescribing rate (APR), for diagnoses which antibiotics are situationally indicated remain unexplored. We assessed the impact of different ICD-10 groupings on outpatient sinusitis APR to characterize its use.

**Methods:**

We included all adult telemedicine and office encounters from January 2021 to March 2022 with an acute or chronic sinusitis ICD-10 from two academic urgent care and eight academic primary care clinics. We calculated an APR (encounters with antibiotics/all encounters) by sinusitis ICD10 code, ICD10 group (acute vs. chronic) and clinic type (Table 1). We then reviewed 100 urgent and 182 randomly selected primary care sinusitis encounters conducted in 2021 to assess symptom documentation and guideline adherence (Table 2). This quality improvement project was deemed non-human subjects research.

**Figure:**
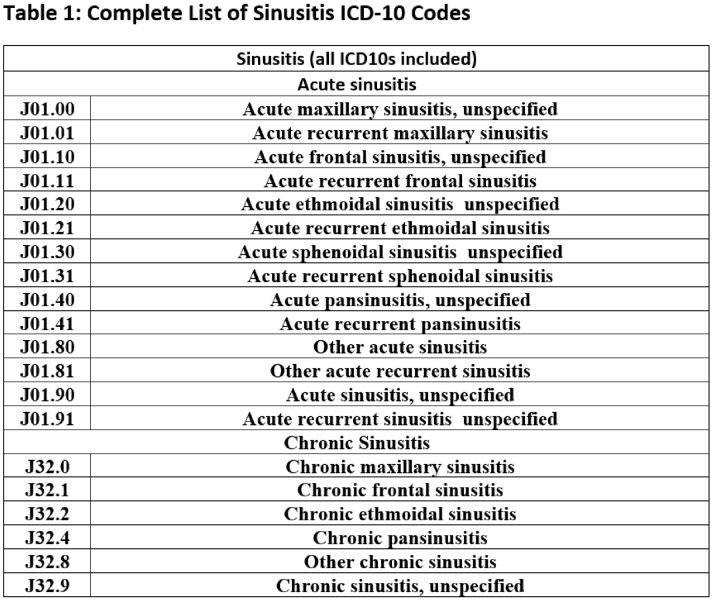

**Figure:**
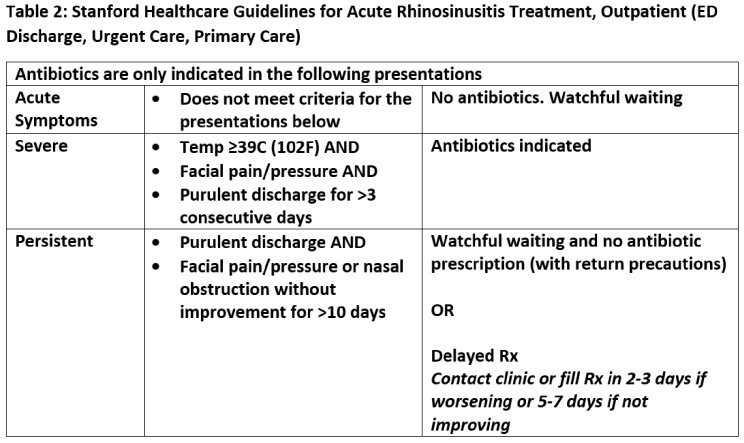

**Results:**

We included 987 sinusitis encounters of 821 unique patients with an overall APR of 56.5% (Table 3). The APR for acute sinusitis encounters was higher than chronic (72.7% vs 22.0%), with a larger difference in primary care (65.5% vs 10.4%) compared to urgent care (76.9% vs 54.2%). Upon chart review, 42.3% (77/182) of primary care sinusitis encounters had no active symptoms (60/77) or did not address sinusitis (17/77); the majority used chronic sinusitis ICD-10s (66/77) (Table 4). Antibiotic prescriptions were guideline adherent for 32.8% of primary care (23/70) compared to 58.0% (40/69) of urgent care encounters (Figure 1).

**Figure:**
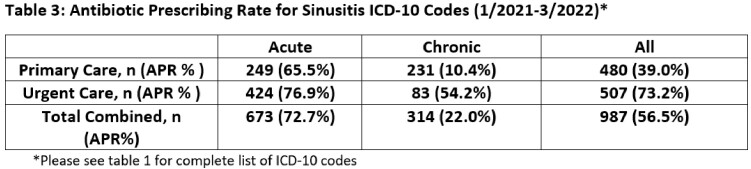

**Figure:**
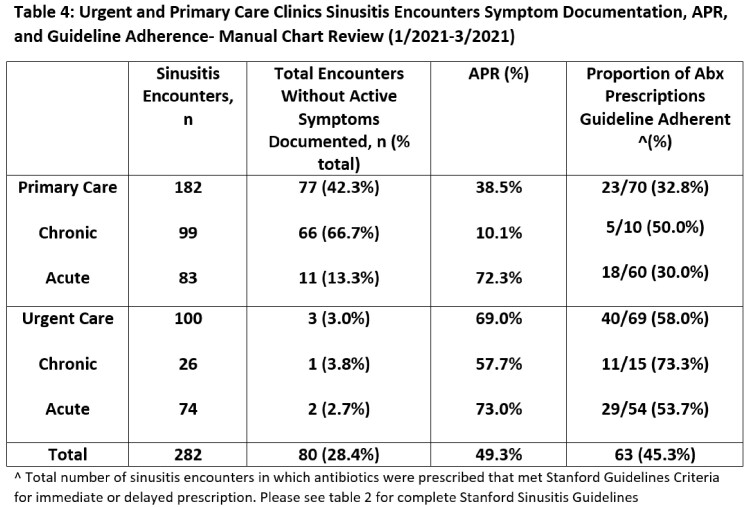

**Figure:**
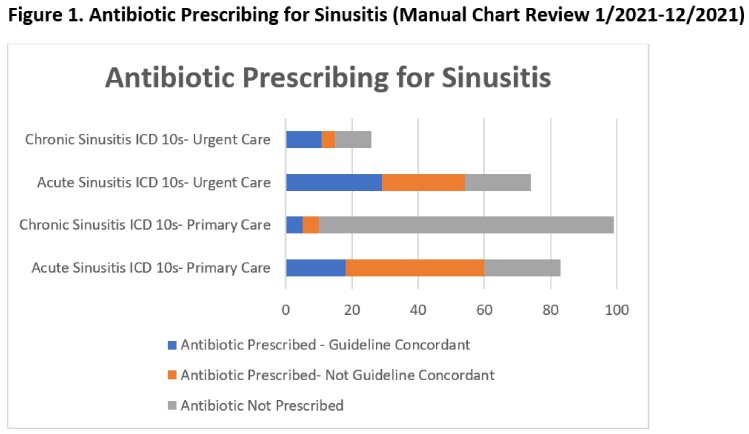

**Conclusion:**

Combining acute and chronic sinusitis ICD10 codes yielded lower APR than acute sinusitis ICD10s alone, predominately in primary care, suggesting a dilution effect. Compared to urgent care, lack of documentation of active symptoms for chronic sinusitis encounters was common in primary care, hindering the assessment of antibiotic appropriateness. Until there is improved ICD10 utilization with methods to control for ‘carryover’ ICD-10s in primary care, acute and chronic sinusitis ICD-10 categories should likely be analyzed separately to increase the usefulness of sinusitis APR as a metric.

**Disclosures:**

**All Authors**: No reported disclosures

